# Village Practice.—III

**Published:** 1918-06-15

**Authors:** 


### VILLAGE PRACTICE.—III.

### An Autobiographical Fragment.

THE two hours between dinner and bed are the most enjoyable of the twenty-four. The mind casts aside its harness, the chafe and fret are forgotten, and, at liberty among his books, a man may browse, fetter-free, like his horse turned out to pasture. On a certain winter evening I had enjoyed this brief spell to the full, reading and meditating beside the fire.

Suddenly the old "grandfather" in the hall commenced to strike ten o'clock, the hour for bed. In a flash my nightly rejuvenation took place. . . .

What awakens most easily in you the memory and renewed experience of days long gone by? Oliver Wendell Holmes, in that most delightful of books "The Autocrat of the Breakfast Table," in dealing with this matter draws attention to a remark often heard, and sometimes met with in books

☞ Previous articles appeared on November 24 and December 22, 1917.

**VILLAGE PRACTICE**—*(continued)*.

such as Bulwer's and in one by Mr. Olmstead. The remark is to the effect that memory, imagination, and old sentiments and associations are more readily reached through the sense of smell than by almost any other channel. Holmes instances, as being particularly operative in his own case, the smell of the marigold. It recalled instantaneously to him an experience of his earliest boyhood, associated with a journey in which he sat securely wedged between the parental buttresses. He saw again the old trap crossing the bridge to the next village-town. It stopped opposite a low, brown, gambrel-roofed cottage from which there emerged one Sally, who, bending over her flower-bed, gathered a "posy" for the little boy. Says he, "Sally lies in the churchyard with a slab of blue slate at her head, lichen-crusted, and leaning a little within the last few years. Cottage, garden beds, posies, grenadier-like rows of seedling onions—stateliest of vegetables—all are gone, but the breath of a marigold brings them all back to me."

To account for this strange connection between the sense of smell and the mind he advances a suggestion which he believed to be new. It was based upon the fact that the olfactory nerve is the only one directly connected with the hemispheres of the brain, the parts in which, as we believe, the intellectual processes are performed.

Whether this be the explanation or not, its ingeniousness will not be disputed, and at all events the intimate relation existing between smell and the reviving of old memories is very noticeable. But, in addition to, and independently of this, there is produced, in my own case, an identical result, by means of the auditory nerve. And on the evening under review there came, with "grandfather's" first stroke of ten, an annihilation of thirty-five years, with all their experiences and results. The man I am, ceased to be. With the wiping out of the years I was once again a little lad in my grandmother's cosy cottage in the Kentish village of Farncroft, four miles from the country town of Bromstead, as Mr. Wells calls it in his book "The New Machiavelli." I was still gazing into the fire, but my own fireside had vanished, dissolved in the melting years, and without a conscious break I was sitting by the roaring wood fire in the old sitting-room. "Grandfather" had "warned" five minutes before, books and toys have been put away, and now the old clock strikes not ten but six—bed-time all the same. It is "Good-night, Marmie" (so I called her). After I have been tucked up I lie wondering why people die; I feel a vague resentment that Marmie should have to sit by the fire alone when I have gone. They tell me Grandad is in Heaven, and, like all children, I cannot easily think of people as dead, but I've seen the grave in the churchyard and that seems very real. How much nicer it would be if people and things just "went on"; if grown people didn't get older, and little boys and girls didn't grow up! It is all such a pity. . . Clang! The vision has gone as quickly as it came. The years have leaped upon me. I am again by my own fireside, my wife has dropped her sewing. The echoes of the old clock striking ten for bed-time have not died away, when into the harmony has crashed that great discord of a doctor's life—the night bell.

No need plaintively to ask to be allowed "just a little longer." It is one of the deep ironies of life that the only human creature who is consumed with a fierce and holy desire to raid a pastrycook's and eat till he is sick is the boy with twopence in his pocket. But away with philosophy now; the ring at the bell means a messenger calling us to practical tasks. . . . Confinement at Easterleigh, nearly nine miles away on the top of the Downs; wife of messenger has been ill, off and on, for twenty-four hours; no progress being made; primipara. A cup of hot cocoa drunk whilst overhauling the bags to make sure nothing is left behind, and we are off. The first five and a half miles of our journey are on the main road. The road is clear and very familiar, and we hum along at a full thirty. On the South Crest, one of the coldest and bleakest bits of road in Wiltshire, we meet a powerful car which passes us at about an equal pace. A glimpse of khaki as we flash past reminds us of the great camps away ahead of us. Scores of thousands of singing cheery boys used to swing along this road in the earlier days of the war. They have passed on. . . . We finish our journey away up on the road that is flung like a wide white ribbon across the undulating summit of the Downs.

We find the patient in charge of a woman, most of whose time, I should imagine, is spent in charing; a most worthy occupation, but one which, unfortunately for us at the present time, does not, *per se*, generate in the person so engaged any obtrusively conspicuous qualifications for sick nursing. However, we are used to this kind of thing in the country, and have to do our best with the material at hand. Examination of the patient reveals an abnormal presentation, which may necessitate serious measures. Having set things going, we may as well take a turn outside, as the night, though cold is clear and bright. All round us is the vast expanse of the Downs. Not a sound is to be heard. We are in a great solitude. Above, with their silent message from Infinity, are the stars—lamps on the highways of God. One other light, and one only, is visible—the lamp in the cottage window. Who was that muttered something about an "anti-climax"? My dear friend, until you have learned to link up and correlate that light in a cottage window to "the last lone star in outer darkness hurled" you will never have touched the hidden spring which unlocks the secret of God and man. Do you remember those words:—

> Lord of all being, throned afar,
> Thy glory flames from sun and star;
> Centre and soul of every sphere,
> Yet to each loving heart how near.

The man who wrote those words was a poet, but for nearly forty years he was also Professor of Anatomy at a great University.

*(To be continued.)*

